# From Stress to Safety: The Chain Mediating Role of Cognitive Reappraisal and Work Engagement in the Influence of Nurses’ Work Stress on Safety Behavior

**DOI:** 10.1155/jonm/7814780

**Published:** 2026-05-27

**Authors:** Kai Zhang, Changchang Chen, Ning Zha, Huan Xu, Chao Wu, Jiayi Zhang, Yifei Wang, Hongjuan Lang

**Affiliations:** ^1^ Department of Nursing, Air Force Medical University, No. 169 Changle West Road, Xi’an, Shaanxi, 710032, China, fmmu.edu.cn

**Keywords:** cognitive reappraisal, safety behavior, stress, work engagement

## Abstract

**Background:**

Nurses face multiple work stress in clinical practice, and the relationship between work stress and safety behavior has attracted much attention. However, no research has explored the chain‐mediating role of cognitive reappraisal and work engagement in this relationship.

**Aim:**

This study aimed to examine the roles of work stress on safety behavior in nurses, along with the mediating effects of cognitive reappraisal and work engagement.

**Methods:**

A cross‐sectional study was conducted among 677 nurses in Shaanxi Province, China, from September to October 2025. The Chinese Nurses Stressor Scale, the Emotion Regulation Questionnaire, the Work Engagement Scale, and the Nurse Safety Behavior Questionnaire were used. IBM SPSS Statistics 26.0 and IBM SPSS AMOS 29.0 were used to analyze data and construct a structural equation model. The research complied with the STROBE checklist.

**Results:**

Nurses’ scores were as follows: work stress (86.09 ± 18.44), cognitive reappraisal (27.75 ± 6.59), work engagement (46.89 ± 13.10), and safety behavior (53.41 ± 6.42). Work stress had a significant negative effect on safety behavior (*r* = −0.200, *p* < 0.001). Cognitive reappraisal and work engagement separately mediated the link between safety behavior and work stress, accounting for 25.12% and 13.95% of the total effect. Furthermore, cognitive reappraisal and work engagement served as chain mediators between work stress and safety behavior, accounting for 7.91% of the total effect.

**Conclusion:**

Work stress among nurses can affect their safety behavior through the mediating role of cognitive reappraisal and work engagement. Future efforts should focus on alleviating nurses’ work stress and cultivating their cognitive reappraisal strategies to enhance work engagement, which may help improve and strengthen nurses’ safety behaviors.

**Implications:**

Nurses need to be fully empowered to ensure patient safety. Hospital managers should prioritize optimizing workload distribution and providing psychological support to reduce nurses’ work stress. Additionally, integrating cognitive reappraisal training into routine nursing education may enhance nurses’ active stress management and engagement.

## 1. Introduction

Patient safety is a core issue in global healthcare. The World Health Organization (WHO) has designated September 17 as World Patient Safety Day to promote global awareness of patient safety [1]. According to the WHO’s Ten Facts on Patient Safety [2], adverse events resulting from unsafe nursing practices are among the top 10 leading causes of death and disability worldwide. In high‐income countries, over 10% of hospitalized patients experienced harm during care, and nearly 50% of these adverse events were preventable. Patient safety is a fundamental principle of healthcare and a cornerstone of continuous quality improvement in medical care [3]. As the healthcare professionals who have the closest contact and most frequent interactions with patients, nurses play a critical role in reducing medical errors and adverse events [4]; their safety behaviors are essential to ensuring patient safety [5]. Nurses’ safety behaviors are the standardized practices performed in clinical care to identify, mitigate, and prevent harm to patients [6, 7]. Previous studies have shown that such behaviors are closely associated with patients’ hospital‐acquired infection rates, in‐hospital mortality rates, rescue failure rates, and healthcare costs [8–11]. Meanwhile, previous studies have also demonstrated that work stress, leadership styles, hospital management systems, work experience, and perceptions of safety culture are key antecedents of nurses’ safe behavior [12–14]. Given nurses’ centrality to patient safety and the robust correlation between nursing safety behavior and patient safety, it is particularly necessary to further investigate the specific factors and underlying mechanisms that enhance safety behavior in nurses. It could provide critical support for intervention practices in the future.

### 1.1. Work Stress and Safety Behavior

Work stress is a key variable influencing safety behavior among nurses [15]. Nurses’ work stress refers to the stress they experience stemming from nursing professional characteristics, specific work content, and nursing‐related work environments [16]. Globally, nurses are facing excessive work stress. In Iran, 60% of nurses experience significant occupational stress [17], while in Ethiopia, 66% face work‐related chronic stress [18]. Nurses are exposed to a complex of multimodal stress [19–21], including physiological strain from overwhelming workloads, psychological exhaustion from occupational trauma, and persistent risks of violence. These factors converge to create a sustained, high‐stress occupational environment. The Conservation of Resource (COR) theory poses that individuals rely on psychological, physiological, and social resources to cope with stress [22]. When nurses face unrelenting high‐level stressors, these resources deplete gradually. Once exhausted, nurses lack the capacity to invest in safety behavior, which requires cognitive and physical effort. This resource depletion ultimately translates to reduced safety performance, such as adhering to protocols or performing procedures carefully. Several studies found a significant negative relationship between stress and safety behavior [15, 23, 24]. However, the specific psychological mechanisms by which work stress diminishes safety behavior have not been fully empirically elucidated, particularly in the context of nursing. Therefore, we proposed Hypothesis 1: Work stress is negatively related to safety behavior in nurses.

### 1.2. The Potential Mediating Role of Emotion Regulation Strategies

Emotion regulation strategies refer to specific methods by which individuals actively and purposefully manage their emotions [25]. Based on the Emotion Regulation Process Interpretation Model [26–28], expression suppression and cognitive reappraisal are two valuable and common strategies that have received considerable empirical attention. Expression suppression is a backward‐looking, repressive regulatory strategy. It maintains superficial emotional stability by inhibiting the external expression of emotions [29]. This strategy continuously depletes substantial psychological resources, exacerbating individuals’ resource exhaustion. In contrast, cognitive reappraisal is a proactive strategy that modulates emotional responses by preemptively interpreting the meaning of emotional events or adjusting personal cognitions regarding such events [30]. This process fosters positive emotional states and promotes adaptive behaviors. Given that nurses engage in sustained patient interactions, prolonged use of expression suppression is more likely to induce occupational burnout [28]. Cognitive reappraisal is more compatible with the sustainability requirements for emotion regulation in nursing settings [31, 32]. Therefore, this study intends to adopt the latter (cognitive reappraisal) as a key variable in this study. Previous studies have demonstrated that cognitive reappraisal mediated the relationship between stress management and behavioral outcomes [28, 33, 34]. This mediation may occur because cognitive reappraisal not only reshapes individuals’ cognitive perspectives on stressful events but also facilitates positive appraisals of stress, modifying how individuals perceive and interpret stressful situations. This mediating effect facilitates the adjustment of adaptive responses, which in turn enhances nurses’ work engagement and work satisfaction. Given that work satisfaction is a key predictor of nurses’ safety behavior [35], cognitive reappraisal may act as a critical variable that influences safety behavior in nurses. Based on the foregoing, Hypothesis 2 was proposed: Cognitive reappraisal mediates the relationship between safety behavior and work stress in nurses.

### 1.3. The Potential Mediating Role of Work Engagement

Work engagement is a key manifestation of nurses’ work‐related attitudes [36], which refers to a positive, enthusiastic work state characterized by high energy, strong focus, and intense dedication during work. It consists of three dimensions: dedication, vigour, and absorption [37]. Numerous studies indicated that work engagement can act as a mediating variable in the field of nursing [38, 39]. According to the COR theory, nurses experiencing high work stress face depletion of personal resources. When resource loss reaches a threshold, nurses may initiate self‐protective behaviors to reduce resource expenditure, which is specifically manifested as reduced work engagement. Previous research has further confirmed that a favorable level of work engagement contributes to safeguarding patient safety, improving job performance, enhancing nursing quality, promoting nurses’ physical health, and boosting work‐related well‐being [35, 40, 41]. Therefore, this study proposes Hypothesis 3: Work engagement mediates the relationship between work stress and safety behavior in nurses.

### 1.4. The Potential Mediating Role of Emotion Regulation Strategies and Work Engagement

The COR theory can be used to explain the relationships between stress, coping strategies, and behavioral outcomes [22]. It is one of the mainstream theories in contemporary social sciences and medical research on stress and positive behavior. It can be simply understood as individuals protecting resources and avoiding resource depletion. That is, when individuals face various external stressors, if the organization can provide them with various resources, or if they themselves possess abundant alternative resources, they can adopt less resource‐depleting behavioral approaches to handle and resolve problems, thereby actively coping with stressors. Work stress is regarded as a source of resource depletion, cognitive reappraisal as a resource replenishment strategy, and work engagement as a positive state following resource accumulation. Nurse safety behavior is defined as behavioral outcomes resulting from resource sufficiency. Therefore, we argue that work stress triggers proactive regulation (cognitive reappraisal) by depleting resources. The resources replenished during this regulatory process further enhance work engagement, ultimately safeguarding safety behavior through accumulated resources. Furthermore, both frequent cognitive reappraisal and high levels of work engagement contribute to increased nurse safety behavior, thereby fully ensuring patient safety. Therefore, this study proposes Hypothesis 4: Work stress influences safety behavior through the chain mediating role of cognitive reappraisal and work engagement.

From the perspective of positive psychology, this study aimed to explore the relationships between work stress, safety behavior, cognitive reappraisal, and work engagement in nurses. It further aimed to examine how work stress influences safety behavior via the independent and chain mediating role of cognitive reappraisal and work engagement. Finally, it sought to provide evidence for hospital administrators to formulate strategies that enhance nurses’ safety behavior and safeguard patient safety.

**FIGURE 1:**
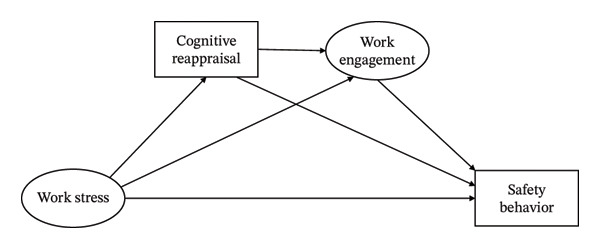
Conceptual framework of the present study.

We proposed this study’s conceptual framework (Figure 1) and hypotheses. Hypothesis 1: Work stress is negatively related to safety behavior in nurses. Hypothesis 2: Cognitive reappraisal is a mediating variable between safety behavior and work stress in nurses. Hypothesis 3: Work engagement is a mediating variable between work stress and safety behavior in nurses. Hypothesis 4: Work stress influences safety behavior through the chain mediating role of cognitive reappraisal and work engagement in nurses.

## 2. Methods

### 2.1. Study Design and Participants

This study employed convenience sampling and a cross‐sectional design, which are widely used to measure the psychological state of the general public. A questionnaire survey was conducted among nurses at four hospitals in Shaanxi Province from September to October 2025. This study was reported in compliance with the Strengthening the Reporting of Observational Studies in Epidemiology (STROBE) guidelines for cross‐sectional studies. Inclusion criteria for nurses were as follows: (1) With a Chinese nurse practicing certificate; (2) with ≥ 1 year of clinical working experience; and (3) with provision of informed consent and voluntary participation in this study. Exclusion criteria for nurses were as follows: (1) Being on leave (including maternity, marriage, or sick leave); (2) being rotating trainees or student nurses; and (3) having experienced significant life changes recently.

Based on the sampling calculation, the sample size ought to be 10 to 20 times the number of research dimensions, with an additional 20% to account for potential attrition [42]. The questionnaire in this study contained 24 dimensions. Consequently, at least 480 nurses were required. In this study, a total of 725 questionnaires were collected. Among these, 15 were completed in less than three minutes, which is nearly impossible to achieve; 14 had inconsistent or obviously illogical personal information; and 19 were from respondents who failed the attention check. The questionnaires mentioned above were excluded to ensure accuracy and validity. Eventually, 677 valid questionnaires were received. The effective response rate was 93.38%.

### 2.2. Data Collection

The questionnaire was distributed using Wenjuanxing, an online survey platform. After obtaining approval from hospital administrators, eligible nurses were informed of the study’s objectives and the voluntary, anonymous nature of their participation. Participants completed the questionnaire by scanning a QR code or clicking a link via smartphone or computer and provided informed consent by clicking the “Agree” button. To ensure data quality, we restricted submissions to one per IP address, required responses to all questions, and included attention check items. Questionnaires with missing data, failed attention checks, or completion times of less than 3 minutes were excluded. The final dataset was independently screened and cross‐verified by two researchers.

### 2.3. Measures

#### 2.3.1. Sociodemographic Questionnaire

Based on previous empirical studies and theories, we designed the sociodemographic questionnaire to collect participants’ basic characteristics and work‐related information (such as age, working experience, gender, educational background, professional title, average monthly salary, and the level of the hospital), which were used as covariates to control for confounding effects.

#### 2.3.2. Chinese Nurses Stressor Scale (CNSS)

The CNSS, that developed by Xiao‐Mei and Liu [43], was used for measurement. This scale has been extensively applied and validated among Chinese nurses [44, 45]. CNSS includes five dimensions: nursing profession and work (7 items), work environment and equipment (3 items), time allocation and workload (5 items), management and interpersonal relationships (9 items), and patient care (11 items). All items are scored with Likert‐4 points, ranging from 1 (Strongly Disagree) to 4 (Strongly Agree). The total score is 35–140. The higher scores indicate more intense stress levels. In this study, the Cronbach’s α of this scale was 0.957, demonstrating high reliability and internal consistency.

#### 2.3.3. Emotion Regulation Questionnaire (ERQ)

The subscale of the Chinese‐adapted ERQ, translated by Wang et al. [46], was utilized to assess cognitive reappraisal ability. ERQ consists of 10 items covering two subscales: expressive suppression (4 items) and cognitive reappraisal (6 items). All items are scored with Likert‐7 points, ranging from 1 (complete disagreement) to 7 (complete agreement). A higher total score under each subscale indicates a higher frequency and tendency to adopt the corresponding emotion regulation strategy in daily life. This study focuses on the cognitive reappraisal strategy of nurses. Given the interactive nature of nursing work, prolonged use of expressive suppression is more likely to lead to occupational burnout among nurses. In contrast, cognitive reappraisal is better suited to the demands of sustained emotional regulation in nursing practice. This subscale has been well validated among Chinese participants [31]. To control for response bias and adhere to standardized testing requirements, this study employed the full‐scale test format, focusing data analysis on the cognitive reappraisal subscale dimension. In this study, the Cronbach’s α of the cognitive reappraisal subscale was 0.921.

#### 2.3.4. Work Engagement Scale

The Work Engagement Scale, developed by Wilmar B. Schaufeli [47], was translated into Chinese by Yiwen Zhang [48]. This scale includes three dimensions of absorption (5 items), dedication (4 items), and vigour (6 items). All items are scored with Likert‐7 points, ranging from 0 (Never) to 6 (Always). The total score is 0–90. The higher the score, the higher the level of the nurses’ work engagement. Cronbach’s α for this scale in this study was 0.909.

#### 2.3.5. Nurse Safety Behavior Questionnaire (NSBQ)

The NSBQ was developed by Shih et al. [49] and translated into Chinese by Rong [50]. The questionnaire is unidimensional with 12 items. All items are scored with Likert‐5 points, ranging from 1 (Never) to 5 (Always). The total score is 12–60. A higher score indicates better performance of nurses in patient safety behavior. In this study, the Cronbach’s α of the scale is 0.907.

### 2.4. Data Analysis

Data analysis was conducted using IBM SPSS Statistics 26.0. Descriptive statistics (frequencies, percentages, means, standard deviations, etc.) were used to describe the sociodemographic characteristics, work stress, safety behavior, cognitive reappraisal, and work engagement. Common Method Bias (CMB) was evaluated using Harman’s one‐way test. Independent‐sample *t*‐test and one‐way ANOVA were used to compare the mean values of safety behavior across different groups. Meanwhile, Pearson correlation analysis was used to assess the relationships among the four variables: work stress, safety behavior, cognitive reappraisal, and work engagement. A chain‐mediating structural equation model was constructed using IBM SPSS AMOS 29.0. A bootstrap procedure (5000 sample repetitions) was applied to evaluate mediation effects (95%*CI*, *p* < 0.05). The model fit was evaluated employing various indices, including the chi‐square/degree of freedom ratio (*χ* ^2^/DF), the adjusted goodness‐of‐fit index (AGFI), Tucker–Lewis index (TLI), the incremental fit index (IFI), root mean square error of approximation (RMSEA), and the comparative fit index (CFI). Cronbach’s α was used to assess the internal consistency reliability of the scales in this study. Statistical significance was defined as a two‐tailed *p* < 0.05.

### 2.5. Ethical Approval

This study was approved by the Ethics Committee of the Second Affiliated Hospital of Air Force Medical University (No. K202509‐01). Participants were provided with detailed information regarding the study objectives, potential risks and benefits, confidentiality safeguards, and their right to withdraw at any time. To ensure anonymity, written consent was waived. Participants indicated their agreement to participate by clicking the “Agree” button at the bottom of the homepage.

## 3. Results

### 3.1. Sociodemographic Features

In this study, a total of 677 nurses were included, among whom 650 (96.00%) were females and 27 (4.00%) were males. Participants had a mean age of 33.75 years (SD = 6.42) and a mean length of working experience of 10.91 years (SD = 6.88). Differences in safety behavior across groups were compared using *t*‐tests and ANOVA. Age, working experience, and professional title showed a statistically significant difference (*p < *0.05). Comprehensive sociodemographic features are presented in Table 1.

**TABLE 1:** Description and univariate analysis of safety behavior (*N* = 677).

Variable	*N* (%)	Safety behavior M (SD)	*t/F*	*p*	Post hoc test
Total	677	53.41 (6.42)			
Gender			−0.580^b^	0.562	
Male	27 (4.00)	52.70 (5.16)			
Female	650 (96.00)	53.44 (6.47)			
Age (years)			6.950^c^	< 0.001	1 < 2, 3, 4
< 26^1^	79 (11.67)	50.20 (8.09)			
26∼35^2^	345 (50.96)	53.46 (6.25)			
36∼45^3^	222 (32.79)	54.22 (5.66)			
> 45^4^	31 (4.58)	55.19 (6.27)			
Marital status			2.440^c^	0.146	
Married	514 (75.92)	53.66 (6.21)			
Unmarried	159 (23.49)	52.53 (7.07)			
Divorced	4 (0.59)	55.50 (2.89)			
Education level			2.750^c^	0.065	
Junior college or lower	64 (9.45)	54.11 (7.01)			
Undergraduate	605 (89.37)	53.38 (6.31)			
Master’s degree or higher	8 (1.18)	48.63 (8.14)			
Working experience (years)			3.876^c^	< 0.005	1 < 4, 1 < 5
< 6^1^	186 (27.47)	51.80 (7.71)			
6∼10^2^	144 (21.27)	53.69 (5.47)			
11∼15^3^	190 (28.07)	53.76 (5.85)			
16∼20^4^	105 (15.51)	54.44 (5.95)			
> 20^5^	52 (7.68)	55.02 (5.73)			
Professional title			5.385^c^	< 0.006	1 < 2
Primary^1^	318 (46.97)	52.56 (7.02)			
Intermediate^2^	305 (0.05)	54.02 (5.54)			
Higher^3^	54 (7.98)	54.93 (6.78)			
Personnel relations			1.146^c^	0.252	
Contract system or Personnel agency system	544 (80.35)	53.55(6.24)			
Bianzhi system^a^	133 (19.65)	52.83(7.10)			
Hospital level			1.600^b^	0.110	
Level III hospital	654 (96.60)	53.48(6.43)			
Level II hospital	23 (3.40)	51.30 (5.78)			
Hospital type			1.242^b^	0.215	
Public Hospital	661 (97.64)	53.45 (6.35)			
Private Hospitals	16 (2.36)	51.44 (8.95)			
Monthly income (RMB)			1.204^c^	0.311	
≤ 4000	114 (16.84)	52.41 (7.42)			
4001–6999	410 (60.56)	53.73 (5.95)			
7000–9999	118 (17.43)	53.53 (6.53)			
≥ 10,000	35 (5.17)	52.49 (7.63)			
Night shift			−1.256^b^	0.210	
Yes	507 (74.89)	53.23 (6.41)			
No	170 (25.11)	53.94 (6.42)			
Weekly work hours (h)			−0.374^b^	0.709	
≤ 40	249 (36.78)	53.29 (6.67)			
> 40	428 (63.22)	53.48 (6.28)			
Departments			0.361^c^	0.925	
Internal medicine	278 (41.06)	53.55 (6.13)			
Surgery	144 (21.27)	53.07 (6.54)			
Obstetrics and gynecology	31 (4.58)	52.87 (5.88)			
Pediatrics	30 (4.43)	54.50 (7.16)			
Emergency	35 (5.17)	53.69 (7.01)			
Intensive care unit	33 (4.87)	52.61 (6.36)			
Operating room	25 (3.69)	54.12 (6.53)			
Others	101 (14.93)	53.31 (6.85)			
Specialist nurse			1.606^b^	0.109	
Yes	214 (31.61)	53.96 (5.89)			
No	463 (68.39)	53.15 (6.64)			

*Note:* The superscript letters 1–5 are designated to compare each sub‐variable when conducting the post hoc test.

^a^Healthcare worker in the bianzhi system can sign a contract without a fixed term with a hospital, benefit from national welfare services, and do not need to worry about losing their job unless they commit a major error.

^b^ *t*‐test.

^c^ANOVA.

### 3.2. CMB Test

This study employed Harman’s one‐way test to assess CMB. An exploratory factor analysis was conducted on all variables from the measurement scales used in this study. The unrotated principal component analysis identified 11 factors with eigenvalues greater than 1, indicating a multifactor structure. The first factor accounted for 24.58% of the variance, which is below the critical threshold of 40%, indicating acceptable commonality [51].

### 3.3. Descriptive Analysis and Correlation Analysis

Among the participants, mean ± SD scores were 86.09 ± 18.44 for work stress, 46.89 ± 13.10 for work engagement, 27.75 ± 6.59 for cognitive reappraisal, and 53.41 ± 6.42 for safety behavior. We found a significant correlation among work stress, work engagement, cognitive reappraisal, and safety behavior in the Chinese nurses. The study revealed that work stress was significantly negatively correlated with cognitive reappraisal (*r* = −0.203, *p* < 0.001), work engagement (*r* = −0.280, *p* < 0.001), and safety behavior (*r* = −0.200, *p* < 0.001). Cognitive reappraisal showed a significant positive correlation with work engagement (*r* = 0.519, *p* < 0.001) and safety behavior (*r* = 0.344, *p* < 0.001). Additionally, work engagement and safety behavior were significantly positively correlated (*r* = 0.309, *p* < 0.001). For further details, please refer to Table 2.

**TABLE 2:** Correlation analysis between work stress, cognitive reappraisal, work engagement, and safety behavior (*N* = 677).

	Variables	Total score range	M(SD)	1	2	3	4	5	6	7	8	9	10	11	12
1	Work stress	35–140	86.09 (18.44)	1											
2	WS1	7–28	18.86 (4.02)	0.829^∗∗∗^	1										
3	WS2	5–20	13.55 (3.54)	0.839^∗∗∗^	0.719^∗∗∗^	1									
4	WS3	3–12	6.36 (2.03)	0.696^∗∗∗^	0.510^∗∗∗^	0.538^∗∗∗^	1								
5	WS4	11–44	27.23 (6.04)	0.909^∗∗∗^	0.650^∗∗∗^	0.687^∗∗∗^	0.581^∗∗∗^	1							
6	WS5	9–36	20.08 (5.84)	0.896^∗∗∗^	0.644^∗∗∗^	0.649^∗∗∗^	0.570^∗∗∗^	0.768^∗∗∗^	1						
7	Work engagement	0–90	46.89 (13.10)	−0.280^∗∗∗^	−0.275^∗∗∗^	−0.265^∗∗∗^	−0.182^∗∗∗^	−0.223^∗∗∗^	−0.241^∗∗∗^	1					
8	Vigour	0–36	19.04 (5.63)	−0.268^∗∗∗^	−0.267^∗∗∗^	−0.232^∗∗∗^	−0.171^∗∗∗^	−0.223^∗∗∗^	−0.233^∗∗∗^	0.938^∗∗∗^	1				
9	Dedication	0–24	13.37 (3.84)	−0.271^∗∗∗^	−0.268^∗∗∗^	−0.256^∗∗∗^	−0.172^∗∗∗^	−0.205^∗∗∗^	−0.244^∗∗∗^	0.895^∗∗∗^	0.775^∗∗∗^	1			
10	Absorption	0–30	14.48 (4.81)	−0.232^∗∗∗^	−0.222^∗∗∗^	−0.244^∗∗∗^	−0.159^∗∗∗^	−0.182^∗∗∗^	−0.189^∗∗∗^	0.910^∗∗∗^	0.766^∗∗∗^	0.730^∗∗∗^	1		
11	Cognitive reappraisal	6–42	27.75 (6.59)	−0.203^∗∗∗^	−0.164^∗∗∗^	−0.156^∗∗∗^	−0.144^∗∗∗^	−0.179^∗∗∗^	−0.199^∗∗∗^	0.519^∗∗∗^	0.530^∗∗∗^	0.428^∗∗∗^	0.452^∗∗∗^	1	
12	Safety behavior	12–60	53.41 (6.42)	−0.200^∗∗∗^	−0.151^∗∗∗^	−0.102^∗∗∗^	−0.175^∗∗∗^	−0.167^∗∗∗^	−0.232^∗∗∗^	0.309^∗∗∗^	0.321^∗∗∗^	0.279^∗∗∗^	0.243^∗∗∗^	0.344^∗∗∗^	1

Abbreviation: WS, work stress.

^∗∗∗^ *p* < 0.001.

### 3.4. Analysis of Mediation Effects

Table 3 shows the fit indices of the structural equation model in this study: *χ* ^2^/DF = 2.322, IFI = 0.989, RMSEA = 0.044, TLI = 0.984, AGFI = 0.962, CFI = 0.989. All indicators fall within reasonable ranges, indicating that the model fits well. The initial fit of this model already meets requirements, so there is no need to reset the model or adjust its parameters.

**TABLE 3:** Fitness indexes of the structural equation model.

Index	*χ* ^2^/DF	RMSEA	AGFI	IFI	TLI	CFI
Criteria	1–3	< 0.08	> 0.8	> 0.9	> 0.9	> 0.9
Actual value	2.322	0.044	0.962	0.989	0.984	0.989

*Note:* *χ* ^2^, chi‐square.

Abbreviations: AGFI, adjusted goodness‐of‐fit index; CFI, comparative fit index; DF, degrees of freedom; IFI, the incremental fit index; RMSEA, root mean square error of approximation; TLI, Tucker–Lewis index.

**FIGURE 2:**
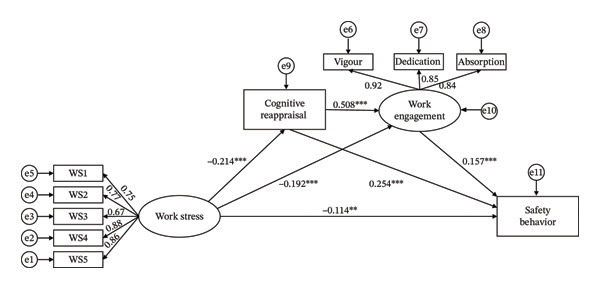
The chain‐mediating model of work stress, cognitive reappraisal, work engagement, and safety behavior. Note: ^∗∗^ *p* < 0.01, ^∗∗∗^ *p* < 0.001, WS: work stress.

The results of the chain mediation model are presented in Table 4 and Figure 2. Work stress’s effect on safety behavior was decomposed into three components: direct effect, indirect effect, and total effect. The direct effect was −0.114, accounting for 53.02% of the total effect. We found that cognitive reappraisal and work engagement had a significant mediating effect, and the total indirect effect value was −0.101, accounting for 46.98% of the total effect (−0.215) of work stress on safety behavior. Specifically, there are three pathways by which mediating effects give rise to indirect effects. First of all, cognitive reappraisal served as a significant mediator in the association between work stress and safety behavior, with a mediating effect value of −0.054, which corresponded to 25.12% of the total effect. Subsequently, work engagement mediated this relationship significantly, yielding a mediating effect of −0.030, which accounted for 13.95% of the total effect. Finally, the chain mediation effect of cognitive reappraisal and work engagement was confirmed, with a combined effect value of −0.017, accounting for 7.91% of the total effect.

**TABLE 4:** The standardized total, direct, and indirect effects of work stress on safety behavior with cognitive reappraisal and work engagement.

Model pathway	β	Bootstraps SE	95% CI		Percent (%)
			Lower	Upper	
Total effect	−0.215	0.037	−0.292	−0.145	100
Direct effect	−0.114	0.037	−0.189	−0.043	53.02
Work stress ⟶ safety behavior					
Indirect effect	−0.101	0.019	−0.142	−0.066	46.98
Work stress ⟶ cognitive reappraisal ⟶ safety behavior	−0.054	0.015	−0.087	−0.030	25.12
Work stress ⟶ work engagement ⟶ safety behavior	−0.030	0.011	−0.058	−0.012	13.95
Work stress ⟶ cognitive reappraisal ⟶ work engagement ⟶ safety behavior	−0.017	0.006	−0.032	−0.007	7.91

## 4. Discussion

This study employed a structural equation model to construct a chain‐mediating model linking work stress, cognitive reappraisal, work engagement, and safety behavior among nurses. All hypotheses tested in our study were supported. The findings provided valuable insights into the complex relationships among these variables, enriching our understanding of the mechanisms linking nursing work stress to safety behavior.

### 4.1. Levels of Work Stress, Safety Behavior, Work Engagement, and Cognitive Reappraisal

Among the participants in this study, the safety behavior score was at a relatively high level, which was consistent with previous studies [6]; however, it was lower than those of emergency department [52] and oncology‐specialized nurses [35]. This discrepancy may be attributed to the following factors: the participants in those studies had strong professional specialization, they mostly dealt with emergency patients (for whom basic information such as infectious disease status is often unknown) or cancer patients (who face a higher risk of safety hazards like self‐harm and self‐injury) [35]. Meanwhile, nurses in those contexts are exposed to occupational risks such as radiation and antineoplastic drugs, which prompts them to enhance their self‐protection awareness and improve their safety behavior.

Age, professional title, and working experience are the main factors for nurses’ low safety behavior. Nurses under 25 years old and those with less than 6 years of working experience scored lower than their elder counterparts, consistent with a previous study [6]. The item with the lowest score was “I pay attention to whether my colleagues comply with safety regulations”. Combined with the results of the univariate analysis, this phenomenon may be related to the fact that junior professional titles accounted for the majority of participants in this study. This group generally has relatively short working experience and low professional ranks; their capabilities are limited to focusing on their own work, leaving them unable to pay attention to whether their colleagues comply with safety regulations. Additionally, there also exists a concern about “offending senior colleagues”.

The mean score of nurses’ work stress in this study was 86.09 ± 18.44 points. Among the dimensions, nursing workload and time allocation received the highest mean score, while the work environment and resources category had the lowest average score. This aligns with previous studies [17, 53], indicating that nurses’ satisfaction with their work environment is improving, but workload and time allocation remain significant practical issues. In addition, the work engagement score in this study was 46.89 ± 13.10, and cognitive reappraisal fell into the moderate range, both of which were consistent with previous studies [16, 31, 32]. These findings imply that there is scope for enhancement in both constructs, and they will continue to be a key aspect of future research.

### 4.2. The Direct Effects of Work Stress

This study found a negative association between work stress and safety behavior, which was consistent with previous studies [23, 54]. Hypothesis 1 was supported. In clinical practice, nurses often face multiple stressors, including high‐intensity, high‐risk work content and complex interpersonal relationships. As roles become increasingly specialized and tasks diversify, the incidence of mental health issues is rising, and work stress is becoming prominent among nurses. A slightly low level of stress can improve work efficiency and serve as a motivator. However, excessive and prolonged stress may weaken nurses’ work enthusiasm, reduce job satisfaction, and trigger adverse nursing events. These consequences further affect the quality of nursing care and pose a threat to patient safety. Therefore, it is essential to emphasize the influence of nurses’ work stress on patient safety. Nursing managers should actively implement the stress management training program, establish an “emotional diary” platform, or regularly organize “peer support group” activities to encourage nurses to vent their stress [55]. At the same time, managers should actively introduce artificial intelligence to monitor the workload and arrange schedules reasonably [56].

### 4.3. The Mediating Effect of Cognitive Reappraisal

This study found that cognitive reappraisal is the mediating variable between work stress and safety behavior. Hypothesis 2 was supported. According to the Emotion Regulation Process Interpretation Model, cognitive reappraisal involves adjusting the cognitive interpretation of events before emotions are generated [34, 57]. Nurses with higher cognitive reappraisal levels, when facing work stress, tend to frame “urgent tasks” as “opportunities for practice” rather than “burdens”, which mitigates the intensity of negative emotions such as anxiety and frustration. However, negative emotions are significant barriers to safety behavior (e.g., anxiety can lead to operational errors). Therefore, cognitive reappraisal enhances nurses’ safety behavior by regulating their cognition of stress. This study shows that the mediating role of cognitive reappraisal accounts for 25.12% of the total effect, indicating that it plays a mediating role in the relationship between safety behavior and work stress.

### 4.4. The Mediating Effect of Work Engagement

This study found that work engagement plays a mediation role in the relationship between work stress and safety behavior among nurses. Hypothesis 3 was confirmed. There is a significant negative correlation between work stress and work engagement, and a decrease in nurses’ work stress usually leads to higher work engagement, which is consistent with previous studies [15, 16, 58]. According to the COR theory, work stress, as a source of resource threat, continuously depletes nurses’ basic psychological and physical resources. This resource depletion leads to a decrease in personal resources. As one of the indicators reflecting the state of personal resources, the improvement of work engagement can prompt nurses to comply with safety norms more rigorously and implement operational details more accurately, thereby enhancing safety behavior levels. Therefore, in high‐pressure situations, implementing interventions such as nursing process optimization and psychological empowerment plays an important role in alleviating the negative effects of stress, promoting nurses’ safety behavior, and safeguarding patient safety.

### 4.5. The Chain Mediating Effect of Cognitive Reappraisal and Work Engagement

This study also found a significant positive correlation between cognitive reappraisal and work engagement among nurses. Work stress could influence nurses’ safety behavior through the chain mediating effect of cognitive reappraisal and work engagement. Hypothesis 4 was confirmed. This supports the applicability of the Emotion Regulation Process Interpretation Model and the COR theory in explaining factors influencing nurses’ safety behavior. According to the COR theory, work stress depletes psychological resources via resource threats, inducing negative emotions and resource loss that ultimately weaken safety behavior motivation. The Emotion Regulation Process Interpretation Model indicates that cognitive reappraisal reduces perceived stress threats by reframing cognitive appraisals of stressful events, thereby interrupting the resource depletion spiral proposed by the COR theory and preserving psychological resources. Preserved resources can mitigate burnout and, through the positive emotions generated by cognitive reappraisal, be transformed into resource gains, which in turn improve nurses’ work commitment and proactive engagement, thus enhancing work engagement. Nurses with higher work engagement, equipped with more psychological resources and positive motivation, tend to actively comply with safety norms, carefully implement operations, and effectively reduce safety incidents caused by resource depletion or burnout. To enhance nurses’ safety behavior, nursing managers not only need to alleviate nurses’ stress but also improve nurses’ personal cognitive reappraisal ability and work engagement [35, 59]. Cognitive reappraisal is an effective strategy for managing and alleviating negative emotional experiences. Nursing administrators should proactively conduct group‐based psychotherapy and improve nurses’ cognitive reappraisal skills. However, considering the cost‐effectiveness of implementing it, cognitive reappraisal training can be integrated into the future nurse orientation training system or daily mental health support system. In particular, nursing managers should pay attention to the group of nurses with low cognitive reappraisal ability and actively guide nurses to focus on the positive aspects of work stress, thereby improving nurses’ work satisfaction and work engagement.

### 4.6. Limitations

This study has the following limitations: firstly, this study employed convenience sampling, and most participants were from Shaanxi Province, which limited the representativeness of the sample. In the future, randomized sampling methods can be used to conduct multiregional and multicentral surveys to improve the generalizability of the findings. Secondly, this study was a cross‐sectional study, which may not be able to clarify the causal relationships between variables. Therefore, longitudinal studies should be adopted in the future. Thirdly, all items in this study were self‐reported by nurses. Future research could consider conducting interviews with nurses and collecting data through a combination of subjective and objective methods to improve the reliability of the results. Fourthly, this study focused on the impacts of cognitive reappraisal strategies. Subsequent studies should consider integrating additional emotion regulation strategies to offer a more holistic understanding of emotional management processes. Lastly, this study may not have fully accounted for potential confounding variables such as shift patterns and workload variability, which could bias the observed associations. Future research may further explore these factors to inform strategies for optimizing nurses’ safety behaviors.

## 5. Conclusion

This study investigated the work stress, safety behavior, cognitive reappraisal, and work engagement of nurses. It indicates that work stress has a significant negative impact on safety behavior among nurses. Cognitive reappraisal and work engagement not only play independent mediating roles but also act as chain mediating variables between work stress and safety behavior. Briefly, these findings provide practical guidance for nursing managers to formulate corresponding management and education measures. Meanwhile, they establish a basis for future research on nurses’ safety behavior and offer theoretical support for developing strategies to alleviate work stress, enhance cognitive reappraisal, and boost work engagement.

## Author Contributions

All authors have made significant contributions to the design and implementation of this research. Kai Zhang: conceptualization, methodology, formal analysis, writing–original draft, and writing–review and editing; Changchang Chen: conceptualization, methodology, data collection, writing–original draft, and writing–review and editing; Ning Zha: methodology, data analysis, and writing–original draft; Huan Xu: formal analysis, visualization, and writing–original draft; Chao Wu: formal analysis, and writing–review and editing; Jiayi Zhang: visualization and writing–original draft; Yifei Wang: conceptualization, methodology, supervision, and writing–review and editing; Hongjuan Lang: conceptualization, supervision, data collection, project administration, funding acquisition, and writing–review and editing.

## Funding

This work was supported by the Research and Practice Project for Comprehensive Reform of Postgraduate Education in Shaanxi Province (grant numbers YJSZG2025187), the Joint Fund Project of the Scientific and Technological Research Project on Major Issues in Military and Aviation Medicine (grant number 2023JSYX19) and the Joint Fund Project of the “Xijing Innovation Research Institute” of the Department of Nursing (grant number LHJJ24HL01).

## Conflicts of Interest

The authors declare no conflicts of interest.

## Data Availability

The data used to support the findings of this study are available from the corresponding authors upon reasonable request.

## References

[1] World Patient Safety Day Campaign [EB/OL], 2025, https://www.who.int/campaigns/world-patient-safety-day.

[2] 10 Facts on Patient safety[EB/OL], 2025, https://www.who.int/news-room/photo-story/detail/10-facts-on-patient-safety.

[3] Patient Safety [EB/OL], 2025, https://www.who.int/health-topics/patient-safety#tab=tab_1.

[4] Samuriwo R. , Bullock A. , Webb K. , and Monrouxe L. V. , Nurses Whisper.’ Identities in Nurses’ Patient Safety Narratives of nurse-trainee Doctors’ Interactions, Medical Education. (2021) 55, no. 12, 1394–1406, 10.1111/medu.14575. 34060110

[5] Scott G. , Hogden A. , Taylor R. , and Mauldon E. , Exploring the Impact of Employee Engagement and Patient Safety, International Journal for Quality in Health Care. (2022) 34, no. 3, 10.1093/intqhc/mzac059. PMC9384574 35899827

[6] Subramaniam C. , Johari J. , Mashi M. S. , and Mohamad R. , The Influence of Safety Leadership on Nurses’ Safety Behavior: the Mediating Role of Safety Knowledge and Motivation, Journal of Safety Research. (2023) 84, 117–128, 10.1016/j.jsr.2022.10.013. 36868640

[7] Yousef A. , Abu Farha R. , and Da’Meh K. , Medication Administration Errors: Causes and Reporting Behaviours from Nurses Perspectives, International Journal of Clinical Practice. (2021) 75, no. 10, 10.1111/ijcp.14541. 34132004

[8] Bressan V. , Mio M. , and Palese A. , Nursing Handovers and Patient Safety: Findings from an Umbrella Review, Journal of Advanced Nursing. (2020) 76, no. 4, 927–938, 10.1111/jan.14288. 31815307

[9] Lim S. H. , Bouchoucha S. L. , Aloweni F. , and Bte Suhari N. A. , Evaluation of Infection Prevention and Control Preparedness in Acute Care Nurses: Factors Influencing Adherence to Standard Precautions, Infect Dis Health. (2021) 26, no. 2, 132–138, 10.1016/j.idh.2020.11.005. 33317963 PMC7833319

[10] Vaismoradi M. , Tella S. , A Logan P. , Khakurel J. , and Vizcaya-Moreno F. , Nurses’ Adherence to Patient Safety Principles: a Systematic Review, International Journal of Environmental Research and Public Health. (2020) 17, no. 6, 10.3390/ijerph17062028. PMC7142993 32204403

[11] Lasater K. B. , Aiken L. H. , Douglas C. et al., Minimum Nurse Staffing Policy Intervention in Queensland Australia Improved Nurse Wellbeing and Patient Safety: a quasi-experimental Intervention Study, International Journal of Nursing Studies. (2025) 171, 10.1016/j.ijnurstu.2025.105178. PMC13248937 40845827

[12] Habibi S. O. O. L. A. A. , Ajri-Khameslou M. , Mirzaei A. , and Bahari Z. , Predictors of Patient Safety Competency Among Emergency Nurses in Iran: a cross-sectional Correlational Study, BMC Health Services Research. (2022) 22, no. 1, 10.1186/s12913-022-07962-y. PMC9036733 35462540

[13] Majdabadi M. A. , Yazdanirad S. , Yarahmadi R. , Abolghasemi J. , and Ebrahimi H. , The Impact of Emotional Intelligence and Personality Traits on the Occurrence of Unsafe Behaviors and Needle Stick Injuries Among the Nurses, Heliyon. (2022) 8, no. 6, 10.1016/j.heliyon.2022.e09584. PMC9344315 35928434

[14] Yan J. , Li L. , Li J. et al., Stepwise Interactive Situated Training Program for Young Nurses’ Safety Behavior and Interrupted Coping Behavior, Healthcare (Basel). (2022) 10, no. 7, 10.3390/healthcare10071157. PMC9320381 35885683

[15] Liu M. , Liu L. , Lv Z. , Ma F. , Mao Y. , and Liu Y. , Effects of Burnout and Work Engagement in the Relationship Between self-efficacy and Safety behaviours-A Chained Mediation Modelling Analysis, Journal of Advanced Nursing. (2024) 80, no. 4, 1473–1483, 10.1111/jan.15925. 37904573

[16] Abou Zeid M. G. , Khedr M. A. , Rayan H. N. , mostafa B. , and El-Ashry A. M. , The Relationship Between Organizational Dehumanization and Work Engagement: the Mediating Effect of Nurses’ Work Stress, BMC Nursing. (2024) 23, no. 1, 10.1186/s12912-024-01841-z. PMC10958847 38515082

[17] Fallahchai R. , Occupational Stress, Dyadic Adjustment and Quality of work-life in Married Nurses: Moderating Effects of Dyadic Coping, International Journal of Nursing Practice. (2022) 28, no. 1, 10.1111/ijn.13032. 34935250

[18] Baye Y. , Demeke T. , Birhan N. , Semahegn A. , and Birhanu S. , Nurses’ work-related Stress and Associated Factors in Governmental Hospitals in Harar, Eastern Ethiopia: a cross-sectional Study, PLoS One. (2020) 15, no. 8, 10.1371/journal.pone.0236782. PMC7398531 32745142

[19] Muhamad Robat R. , Mohd Fauzi M. F. , Mat Saruan N. A. , Mohd Yusoff H. , and Harith A. A. , Why so Stressed? A Comparative Study on Stressors and Stress Between Hospital and Non-hospital Nurses, BMC Nursing. (2021) 20, no. 1, 10.1186/s12912-020-00511-0. PMC7780689 33390159

[20] Ye L. , Xu Y. , Cui L. et al., The Relationship Between Occupational Stressors and Resilience Among Emergency Department Nurses: a Multicenter Cross-Sectional Network Analysis, Journal of Advanced Nursing. (2025) 10.1111/jan.70317. 41157805

[21] Chen Y. , Wu H. , Ho J. , Cheng N. Y. , Guo Y. L. , and Shiao J. S. C. , Exploring the Association Between Patient-Nurse Ratio and Nurse’ Occupational Stressors: a Cross-Sectional Study, Journal of Nursing Management. (2025) 2025, no. 1, 10.1155/jonm/6160674. PMC12126261 40454001

[22] Hobfoll S. E. , Conservation of Resources. A New Attempt at Conceptualizing Stress, American Psychologist. (1989) 44, no. 3, 513–524, 10.1037/0003-066x.44.3.513, 2-s2.0-0024632747. 2648906

[23] Man S. S. , Wang D. , Tsang S. N. H. , Liu L. , and Chan A. H. S. , Relationships Between Occupational Stress and Occupational Safety and Health Outcomes Amongst Construction Workers: a meta-analysis of Evidence from the past Twenty Years, Safety Science. (2025) 191, 10.1016/j.ssci.2025.106939.

[24] Hou M. , Wei W. , Wang S. et al., Challenge-Hindrance Stressors and Novice Nurses Safety Behaviour: the Mediating Role of Regulatory Focus and the Moderating Role of Workplace Spirituality, Journal of Advanced Nursing. (2024) . 10.1111/jan.16723 39740076

[25] Van Beveren M. , De Clercq B. , and Braet C. , Just the Way You are. Understanding Emotion Regulation Strategies in Youth from Temperamental Differences, Journal of Research in Personality. (2020) 88, 10.1016/j.jrp.2020.103989.

[26] Cavicchioli M. , Scalabrini A. , Northoff G. , Mucci C. , Ogliari A. , and Maffei C. , Dissociation and Emotion Regulation Strategies: a meta-analytic Review, Journal of Psychiatric Research. (2021) 143, 370–387, 10.1016/j.jpsychires.2021.09.011. 34592484

[27] Khan A. J. , Maguen S. , Straus L. D. , Nelyan T. , Gross J. , and Cohen B. , Expressive Suppression and Cognitive Reappraisal in Veterans with PTSD: Results from the Mind Your Heart Study, Journal of Affective Disorders. (2021) 283, 278–284, 10.1016/j.jad.2021.02.015. 33578339

[28] Gross J. J. and John O. P. , Individual Differences in Two Emotion Regulation Processes: Implications for Affect, Relationships, and well-being, Journal of Personality and Social Psychology. (2003) 85, no. 2, 348–362, 10.1037/0022-3514.85.2.348, 2-s2.0-0242474299. 12916575

[29] Ma X. , Xiao Z. , Chen W. , and Zhao S. Y. , Deciphering the Psychological Tapestry of Fgids: Unveiling the Impact of Negative Affect, Rumination, and Expression Suppression, BMC Public Health. (2025) 25, no. 1, 10.1186/s12889-024-21205-1. PMC11720358 39789461

[30] Stover A. D. , Shulkin J. , Lac A. , and Rapp T. , A meta-analysis of Cognitive Reappraisal and Personal Resilience, Clinical Psychology Review. (2024) 110, 10.1016/j.cpr.2024.102428. 38657292

[31] Huang H. , Su Y. , Liao L. , Li R. , and Wang L. , Perceived Organizational Support, self-efficacy and Cognitive Reappraisal on Resilience in Emergency Nurses Who Sustained Workplace Violence: a Mediation Analysis, Journal of Advanced Nursing. (2024) 80, no. 6, 2379–2391, 10.1111/jan.16006. 38050872

[32] Bai B. and Bai C. , Strength Use and Thriving at Work Among Chinese Nurses: the Mediating Roles of Control Beliefs About Stress and Cognitive Reappraisal, Journal of Nursing Management. (2024) 2024, no. 1, 10.1155/2024/5509059. PMC11918909 40224778

[33] Hamza J. , Vytykacova S. , Jansakova K. , and Rajčáni J. , Cognitive Reappraisal and Acceptance Following Acute Stress, Stress and Health. (2024) 40, no. 5, 10.1002/smi.3469. 39229764

[34] Marciniak M. A. , Homan S. , Zerban M. et al., Positive Cognitive Reappraisal Flexibility is Associated with Lower Levels of Perceived Stress, Behaviour Research and Therapy. (2024) 183, 10.1016/j.brat.2024.104653. 39536535

[35] Ma F. , Zhu Y. , Liu L. , and Chen H. , Work Engagement and Safety Behavior of Nurses in Specialized Cancer Hospitals: the Mediating Role of Self-Efficacy, Journal of Nursing Management. (2023) 2023, 9034073–9034079, 10.1155/2023/9034073. 40225692 PMC11919182

[36] Wu C. , Cheng S. , Wu J. et al., Factors Influencing Work Engagement Among Male Nurses: a Structural Equation Model, Nursing Open. (2023) 10, no. 12, 7749–7758, 10.1002/nop2.2016. 37794567 PMC10643843

[37] Calvo J. M. , Kwatra J. , Yansane A. , Tokede O. , Gorter R. C. , and Kalenderian E. , Burnout and Work Engagement Among US Dentists, Journal of Patient Safety. (2021) 17, no. 5, 398–404, 10.1097/pts.0000000000000355, 2-s2.0-85021834613. 28671911

[38] Cai Y. , Li Q. , Cao T. , and Wan Q. , Nurses’ Work Engagement: the Influences of Ambidextrous Leadership, Clinical Nurse Leadership and Workload, Journal of Advanced Nursing. (2023) 79, no. 3, 1152–1161, 10.1111/jan.15086. 34723406

[39] Alluhaybi A. , Wilson A. , Usher K. , and Durkin J. , Impact of Nurse Manager Leadership Styles on Work Engagement: a Systematic Literature Review, Journal of Nursing Management. (2023) 2023, 5090276–5090279, 10.1155/2023/5090276. 40225613 PMC11918969

[40] Midje H. H. , Nyborg V. N. , Nordsteien A. , Øvergård K. I. , Brembo E. A. , and Torp S. , Antecedents and Outcomes of Work Engagement Among Nursing Staff in long-term Care facilities-A Systematic Review, Journal of Advanced Nursing. (2024) 80, no. 1, 42–59, 10.1111/jan.15804. 37519065

[41] Waltz L. A. , Munoz L. , Weber J. O. H. N. S. O. N. H. , and Rodriguez T. , Exploring Job Satisfaction and Workplace Engagement in Millennial Nurses, Journal of Nursing Management. (2020) 28, no. 3, 673–681, 10.1111/jonm.12981. 32068932

[42] Preacher K. J. and Kelley K. , Effect Size Measures for Mediation Models: Quantitative Strategies for Communicating Indirect Effects, Psychological Methods. (2011) 16, no. 2, 93–115, 10.1037/a0022658, 2-s2.0-79958851756. 21500915

[43] Xiao-Mei Li and Liu Y.-jun , Job Stressors and Burnout Among Staff Nurses, Chinese Journal of Nursing. (2000) 35, no. 11, 645–649.

[44] Liang N. , Zhao J. , Ren J. et al., The Mediating Effect of Positive Psychological Capital Between Nurses Spare Time Planning and Occupational Stress, Journal of Nursing Science. (2025) 40, no. 03, 82–85, 10.3870/j.issn.1001-4152.2025.03.082.

[45] Tian D. , Liu N. , Liu Y. et al., Tools for Assessing Perceived Stress in Nursing Staff:A Literature Review, Journal of Nursing Science. (2022) 37, no. 22, 18–22, 10.3870/j.issn.1001-4152.2022.22.018.

[46] Wang Li , Liu H. , Li Z. et al., Reliability and Validity of Emotion Regulation Questionnaire Chinese Revised Version, China Journal of Health Psychology. (2007) no. 06, 503–505, 10.3969/j.issn.1005-1252.2007.06.034.

[47] Schaufeli W. B. , Salanova M. , González-Romá V. , and Bakker A. B. , The Measurement of Engagement and Burnout: a Two Sample Confirmatory Factor Analytic Approach, Journal of Happiness Studies. (2002) 3, no. 1, 71–92, 10.1023/a:1015630930326.

[48] Zhang Y. and Gan Y. , The Chinese Version of Utrecht Work Engagement Scale: an Examination of Reliability and Validity, Chinese Journal of Clinical Psychology. (2005) no. 03, 268–270, 10.3969/j.issn.1005-3611.2005.03.005.

[49] Shih C. , Chang L. , Chen J. et al., The Factors Influencing Safety Behavior of Medical Staffs in Emergency Room of a Medical Center in Taiwan, Journal of Management. (2008) 25, no. 4, 451–465.

[50] Rong Y. , The Relationship Between Patient Safety Culture and Safety Behavior, 2009, Tzu Chi University.

[51] Ghasemi A. and Zahediasl S. , Normality Tests for Statistical Analysis: a Guide for Non-statisticians, The Internet Journal of Endocrinology. (2012) 10, no. 2, 486–489, 10.5812/ijem.3505, 2-s2.0-84864023133. PMC3693611 23843808

[52] Zhang A. , Ye L. , Feng X. et al., A Study on the Current Status and Correlation Between Patient Safety Culture Perceptions and Nurse Safety Behaviors in the Emergency Department of a Tertiary Care Hospital, Journal of Nurses Training. (2022) 37, no. 13, 1225–1230, 10.16821/j.cnki.hsjx.2022.13.015.

[53] Dziedzic B. , Lodziana K. , Marcysiak M. , and Kryczka T. , Occupational Stress and Social Support Among Nurses, Frontiers in Public Health. (2025) 13, 10.3389/fpubh.2025.1621312. PMC12213681 40606078

[54] Hou M. , Wei W. , Wang S. , Hu J. , and Chen Y. , Challenge-Hindrance Stressors and Novice Nurses’ Safety Behaviour: the Mediating Role of Regulatory Focus and the Moderating Role of Workplace Spirituality, Journal of Advanced Nursing. (2025) 81, no. 11, 7597–7608, 10.1111/jan.16723. 39740076

[55] Aust B. , Leduc C. , Cresswell-Smith J. et al., The Effects of Different Types of Organisational Workplace Mental Health Interventions on Mental Health and Wellbeing in Healthcare Workers: a Systematic Review, International Archives of Occupational and Environmental Health. (2024) 97, no. 5, 485–522, 10.1007/s00420-024-02065-z. 38695906 PMC11130054

[56] Gonzalez-Garcia A. , Perez-Gonzalez S. , Benavides C. , Pinto-Carral A. , Quiroga-Sánchez E. , and Marqués-Sánchez P. , Impact of Artificial Intelligence-Based Technology on Nurse Management: a Systematic Review, Journal of Nursing Management. (2024) 2024, no. 1, 10.1155/2024/3537964. PMC11919197 40224848

[57] Zhang Y. , Liu F. , Ma J. et al., Psychological Stress and Depression Symptoms in Nursing Undergraduates: the Chain Mediating Effect of Cognitive Reappraisal and Ruminate Thinking, BMC Nursing. (2025) 24, no. 1, 10.1186/s12912-024-02604-6. PMC11702140 39762876

[58] Cabrera-Aguilar E. , Zevallos-Francia M. , Morales-Garcia M. et al., Resilience and Stress as Predictors of Work Engagement: the Mediating Role of self-efficacy in Nurses, Frontiers in Psychiatry. (2023) 14, 10.3389/fpsyt.2023.1202048. PMC10464840 37649562

[59] Huang Y. , Spiritual Leadership and Job Engagement: the Mediating Role of Emotion Regulation, Frontiers in Psychology. (2022) 13, 10.3389/fpsyg.2022.844991. PMC9046577 35496230

